# Reduced basal forebrain atrophy progression in a randomized Donepezil trial in prodromal Alzheimer’s disease

**DOI:** 10.1038/s41598-017-09780-3

**Published:** 2017-09-15

**Authors:** Enrica Cavedo, Michel J. Grothe, Olivier Colliot, Simone Lista, Marie Chupin, Didier Dormont, Marion Houot, Stephane Lehéricy, Stefan Teipel, Bruno Dubois, Harald Hampel, Hippocampus Study Group, Bernard Croisile, Guy Louis Tisserand, Alain Bonafe, Pierre J Ousset, Olivier Rouaud, Fréderic Ricolfi, Alain Vighetto, Florence Pasquier, Christine Delmaire, Mathieu Ceccaldi, Nadine Girard, Françoise Duveau, Marie Sarazin

**Affiliations:** 1 AXA Research Fund & UPMC Chair, Paris, France; Sorbonne Universities, Pierre et Marie Curie University, Paris 06, Institute of Memory and Alzheimer’s Disease (IM2A) & Brain and Spine Institute (ICM) UMR S 1127, Department of Neurology, Hopital Pitié-Salpêtrière, Paris, France; 2 Laboratory of Alzheimer’s Neuroimaging and Epidemiology, IRCCS Centro San Giovanni di Dio Fatebenefratelli, Brescia, Italy; 3 German Center for Neurodegenerative Diseases (DZNE) – Rostock/Greifswald, Rostock, Germany; 4 Department of Psychosomatic Medicine, University of Rostock, Rostock, Germany; 5 Sorbonne Universités, UPMC Univ Paris 06, Inserm, CNRS, Institut du cerveau et la moelle (ICM), APHP- Hôpital Pitié-Salpêtrière, Paris, France; 6 Inria, Aramis project-team, Centre de Recherche de, Paris, France; 7 CATI – Centre d’Acquisition et de Traitement des Images, Paris, France; 8 AP-HP, Neuroradiology Department, Hôpital de la Pitié-Salpêtrière, Paris, France; 9 Institute of Memory and Alzheimer’s Disease (IM2A), Centre of Excellence of Neurodegenerative Disease (CoEN), ICM, APHP Department of Neurology, Hopital Pitié-Salpêtrière, University Paris 6, Paris, France; 10 CENIR, Hôpital de la Salpêtriere, Paris, France; 11 INSERM U1127, Institut du Cerveau et de la Moelle Epiniere (ICM), Paris, France; 12 Sorbonne Universites, Universite Pierre et Marie Curie-Paris 6, Paris, France; 13 Sorbonne Universities, Pierre et Marie Curie University, Paris 06, Institute of Memory and Alzheimer’s Disease (IM2A) & Brain and Spine Institute (ICM) UMR S 1127, Department of Neurology, Hopital Pitié-Salpêtrière, Paris, France; 14 Department of Neuropsychology, Hôpital Neurologique Pierre Wertheimer, Lyon, France; 15 Hôpital Neurologique Pierre Wertheimer, Lyon, France; 16 CHRU, Gui de Chauliac, Montpellier, France; 17 Centre Mémoire/Centre de Recherche Clinique – Gérontopôle, Hôpital Casselardit, Toulouse, France; 18 Hôpital General, Dijon, France; 19 Lyon 1 university, Hospices Civils de Lyon, hôpital neurologique, service de neurologie D, Lyon, France; 20 Université de Lille, Inserm U1171, CHU, centre mémoire 59000, Lille, France; 21 Université de Lille, Inserm U1171, Service de Neuroradiologie, Hôpital Roger Salengro, Lille, France; 22 Hôpital de la Timone, Marseille, France; 23 Eisai SAS, La Defense 2, Cedex, France; 24 Neurologie de la Mémoire et du Langage, Université Paris Descartes, Sorbonne Paris Cité, INSERM UMR S894, Centre Hospitalier Sainte Anne, Paris, France

## Abstract

Acetylcholinesterase inhibitors are approved drugs currently used for the treatment of Alzheimer’s disease (AD) dementia. Basal forebrain cholinergic system (BFCS) atrophy is reported to precede both entorhinal cortex atrophy and memory impairment in AD, challenging the traditional model of the temporal sequence of topographical pathology associated with AD. We studied the effect of one-year Donepezil treatment on the rate of BFCS atrophy in prodromal AD patients using a double-blind, randomized, placebo-controlled trial of Donepezil (10 mg/day). Reduced annual BFCS rates of atrophy were found in the Donepezil group compared to the Placebo treated arm. Secondary analyses on BFCS subregions demonstrated the largest treatment effects in the Nucleus Basalis of Meynert (NbM) and the medial septum/diagonal band (Ch1/2). Donepezil administered at a prodromal stage of AD seems to substantially reduce the rate of atrophy of the BFCS nuclei with highest concentration of cholinergic neurons projecting to the cortex (NbM), hippocampus and entorhinal cortex (Ch1/2).

## Introduction

Degeneration of cholinergic neurons in the basal forebrain cholinergic system (BFCS) and the subsequent loss of cholinergic neurotransmission in the cerebral cortex and limbic system are crucial pathophysiological events that trigger the cognitive deterioration observed in patients with Alzheimer’s disease (AD) dementia^1, 2^.

So far, Acetylcholinesterase inhibitors (AChEI) are among the few drugs approved for the treatment of AD^3, 4^. Traditionally, AChEI have been categorized as “symptomatic drugs” since they delay the development of clinical symptoms of AD without previous convincing evidence of affecting hypothesized key disease pathophysiology, characterized by the extracellular deposition of accumulated amyloid beta (Aβ) peptides into amyloid plaques and the intracellular accumulation of neurofibrillary tangles in the brain. However, there is a renewed debate on the involvement of cholinergic therapies in various pathophysiological mechanisms and cascades associated with AD. There is evidence showing that AChEI prevent experimentally induced Aβ brain deposition, suggesting that AChEI may delay the progression of the disease^5, 6^. Indeed, reduced rate of atrophy in grey and white matter volumes and metabolic changes were found in AD dementia patients after AchEI treatment^7^^–^^10^ indicating the potential disease-modifying effect of AchEI. However, conflicting results were reported in clinical trials on mildly cognitively impaired (MCI) subjects^11^^–^^16^, probably due to the substantial degree of clinical heterogeneity that characterizes this target population.

The nucleus basalis of Meynert (NbM) is one of the most vulnerable structures to neurofibrillary degeneration^17^ and represents the principal source of cholinergic innervation of the cerebral cortex and the amygdala^18^. In contrast to its widespread projections to major cortical areas, the NbM receives its main cortical inputs from the piriform, orbitofrontal, medial temporal, entorhinal and anterior cingulate cortices as well as from the anterior insula and temporal pole^18, 19^.

Measurements of BFCS atrophy on high-resolution structural magnetic resonance imaging (MRI) are being used as *in vivo* surrogate markers for cholinergic degeneration. Recent studies could demonstrate *in vivo* BFCS atrophy in AD dementia patients and in prodromal AD individuals^20^^–^^22^, including increased rates of BFCS atrophy in AD and MCI compared to controls during 2 years of follow-up^23^. These changes were found to be most pronounced in the posterior part of the NbM^24^, reflecting the neuropathological literature^25^. Recently published results indicated that the atrophy of NbM may precede and predict atrophy of the entorhinal cortex in relation to different stages of AD pathophysiology^26^. These evidences were in line with previous findings highlighting the existence of a correlation between BFCS atrophy and cortical amyloid burden in asymptomatic at-risk and pre-dementia stages of AD^27^^–^^29^.

Based on the data previously reported by the Hippocampus Study Clinical Trial (NCT00403520)^13, 16^ and the recent literature results suggesting an early involvement of BFCS in AD pathophysiology^26, 28, 30^, the objective of this explorative study is to further investigate the effect of one-year Donepezil treatment on the rate of BFCS atrophy in prodromal AD patients.

## Results

### Study design and clinical results

This study included 332 patients with prodromal AD that participated in the multi-centre double-blind, randomized, placebo-controlled trial with a Donepezil treatment period (10 mg/day) of 12 months (Clinical Trial.gov Number: NCT00403520, Submission Date: November 21, 2006)^13^. From the per-protocol population, we selected the participants with imaging scans with enough quality for the data analysis (Placebo N = 88, Donepezil N = 75). No difference between Donepezil and Placebo groups was present in terms of sociodemographic features (age, gender and education), clinical features (depressive symptoms), global cognition and memory performances (Table 1). Finally, no differences were found in the time period of MRI scans and in the frequencies of scans executed with 3 Tesla MRI machine between groups (Table 1).

**Table 1:** Baseline Demographic and Clinical Characteristics of patients performing baseline and follow-up MRI.

	Placebo (n = 88)	Donepezil (n = 75)	*p-value*	F value (df)
Age, years	73 (6.7)	73 (6.8)	0.930	0.008 (1,161)
Gender				
F/M	46/42	37/38	0.708	
Education, n (%)				
No education	1 (0.01)	0 (0.0)	0.140	
Primary	6 (6.8)	8 (10.6)		
Certificate of Primary	43 (48.8)	26 (34.6)		
Education	13 (14.7)	21 (28)		
Secondary	25 (28.4)	20 (26.6)		
Higher education				
Follow-up MRI (days)	376 (48)	373 (43)	0.730	0.120 (1,161)
FCSRT (Free recall)	11.3 (5.7)	12.1 (5.2)	0.357	0.853 (1,161)
FCSRT (Total recall)	29.4 (10)	31 (8.7)	0.267	1.267 (1,161)
Hamilton Rating Scale for Depression	3.6 (2.8)	3.3 (2.7)	0.492	0.475 (1,161)
ADAS-COG-MCI	12.4 (4.3)	12.3 (4.5)	0.975	0.001 (1,161)
MMSE	25.9 (2.8)	26 (2.2)	0.831	0.046 (1,161)
APOE genotype,				
Positive APOE ε4 n (%)	18 (47%)	16 (59%)	0.344	
Missing n (%)	65 (63%)	86 (24%)		
3 Tesla MRI (%)	23%	29%	0.336	

Means and standard deviations are reported for continuous variables, numbers and percentages for the dichotomous ones. P-values denote significant differences at ANOVA and Chi-square tests. FCSRT = Free and Cued Selective Reminding Test, MRI = Magnetic Resonance Imaging, df = degree﻿s of freedom.

### Grey Matter and BFCS baseline volume features

In order to check baseline brain volume differences between groups, we first examined the baseline BFCS and total grey matter (GM) volume differences, normalized to the total intracranial volume, between Placebo and Donepezil using ANOVA. No differences were found (Table 2). Distributions of GM and BFCS nuclei volumes in each treatment group stratified by MRI field strength at baseline and follow-up scans are represented in the Supplementary Fig. 1.

**Table 2:** Grey Matter and Basal forebrain cholinergic system volumes at baseline.

	Placebo (n = 88)	Donepezil (n = 75)	*p-value*	F value (df)
GM (cm^3^)	535 ± 60	543 ± 60	0.352	0.870 (1,161)
BFCS (mm^3^)	617 ± 125	648 ± 139	0.132	2.296 (1,161)
NbM (mm^3^)	471 ± 100	494 ± 109	0.171	1.889(1,161)
Ch4p (mm^3^)	179 ± 45	184 ± 46	0.482	0.496 (1,161)
Ch1/2 (mm^3^)	109 ± 25	117 ± 28	0.068	3.382 (1,161)

Means and standard deviations are reported, p-values denote significant differences at One-Way ANOVA including age, gender, MRI field strength (1.5 T or 3 T) and MRI scanner manufacturer (Philips, GE Healthcare or Siemens) as covariates. All the Volumes are normalized to the total intracranial volume (TIV).

Abbreviations: BFCS = Basal Forebrain Cholinergic System; NbM = Nucleus Basalis of Meynert; Ch4p = posterior Nucleus basalis Meynert (NBM); Ch1/2 = combined clusters of the medial septum and the vertical limb of the diagonal band of Broca, df= degree﻿s of freedom.

### Donepezil reduced GM and BFCS atrophy

**Figure 1:**
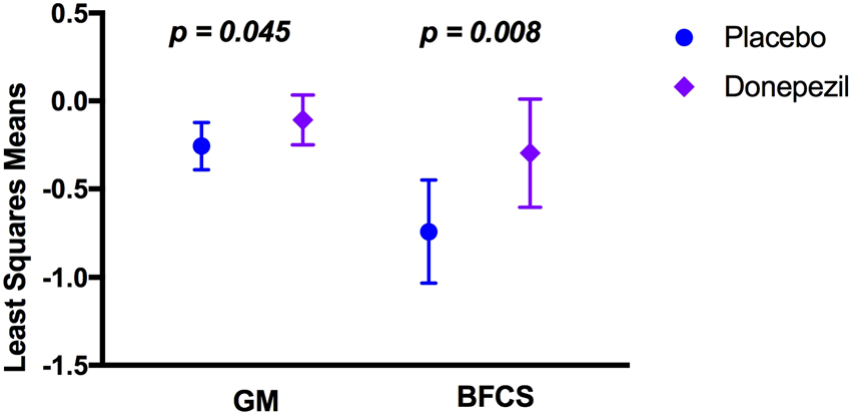
Grey Matter (GM) and Basal Forebrain Cholinergic System (BFCS) Annualized Percentage Change (APC) describing significant atrophy reduction in prodromal AD patients treated one year with Donepezil. Least squares means and 95% Confidence Interval are reported, p-values denote significant differences between treatment groups at the General Linear Model including age, gender, MRI field strength (1.5 T or 3 T) and MRI scanner manufacturer (Philips, GE Healthcare or Siemens) as covariates.

**Figure 2:**
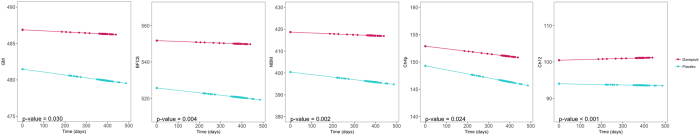
Mean trajectories of Grey Matter and Basal Forebrain Cholinergic System indicating significant volume changes over time, from baseline scan to follow-up scan, as resulted from the linear mixed-effects model.

The main aim of the study was to investigate the effect of Donepezil treatment on brain volume changes involved in the cholinergic circuit over the study period by measuring the BFCS and GM Annualized Percentage Change (APC). Results from the general linear model, including age, gender, MRI field strength as well as MRI scanner manufacturer as covariates, revealed reduced APC of the total GM in the Donepezil group compared to the Placebo (p = 0.045, Fig. 1). A treatment difference of −0.15 percentage points for total GM volume was found. Moreover, the Donepezil group exhibited a lower rate of atrophy of the whole BFCS over a 1-year period compared to the Placebo group (APC −0.30% vs −0.74%, respectively, p = 0.008, Fig. 1) with a treatment difference of −0.44. Supplemented analysis using the linear mixed effect model (Fig. 2) revealed that GM (p = 0.030; estimate: time*donepezil = −0.001, time*placebo = −0.004) and BFCS (p = 0.004; estimate: time*donepezil = −0.005 and time*placebo = −0.013) volumes were significantly decreased in the Placebo group compared with the Donepezil group throughout the 12 months of treatment period.

### Secondary analysis on BFCS subregions

In addition, a secondary analysis was conducted on BFCS subregions. In particular, in those subregions representing the best separable divisions within the BFCS such as the Ch1/2 sector and the NbM, as well as the Ch4p division of the NbM, since previous studies reported that the Ch4p is the most BFCS region affected in AD.

**Figure 3:**
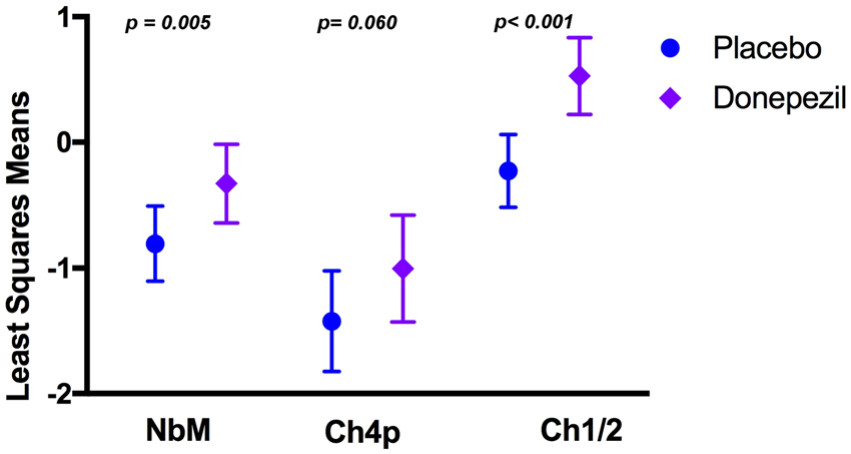
Annualized Percentage Change (APC) of Basal forebrain cholinergic system subregions indicating a specific reduction of trophy after one year of treatment with Donepezil in the Necleus Basalis of Mynert and in the medial septum and the vertical limb of the diagonal band of Broca. Least squares means and 95% Confidence Interval are reported, p-values denote significant differences between treatment groups at General Linear Model including age, gender, MRI field strength (1.5 T or 3 T) and MRI scanner manufacturer (Philips, GE Healthcare or Siemens) as covariates. NbM = Nucleus Basalis of Meynert; Ch4p = posterior Nucleus Basalis Meynert (NBM); Ch1/2 = combined clusters of the medial septum and the vertical limb of the diagonal band of Broca.

Results from the general linear model showed reduced APC in the Donepezil group compared to the Placebo group in the rostromedial nuclei (Ch1/2, p < 0.001, treatment difference = −0.76) as well as in the NbM (p = 0.005 treatment difference = −0.48; defined as the sum of the volume of its subdivisions: Ch4a-i, Ch4al/NSP and Ch4p) (Fig. 2). Finally, a trend of significance between groups was found in the APC of the posterior NbM subregion (Ch4p, p = 0.060, treatment difference = −0.042, Fig. 3). Supplemented analysis using the linear mixed effect model (Fig. 2) revealed that during the 12 months of treatment period, NbM (p = 0.002; estimate: time*donepezil = −0.004, time*placebo = −0.012), Ch1/2 (p < 0.001; estimate: time*donepezil = 0.002, time*placebo = −0.001) and Ch4p volumes were significantly decreased in the Placebo group compared with the Donepezil group (p = 0.024; estimate: time*donepezil = −0.005, time*placebo = −0.007).

## Discussion

Our outcomes build on the hypothesis that AChEI treatment has a relevant influence on the neurodegenerative progression of the BFCS in patients with an amnestic syndrome of the hippocampal type, a clinical high risk manifestation of prodromal AD^31^. Overall, our findings revealed a reduction of total GM and BFCS atrophy in prodromal AD patients treated with Donepezil compared to the Placebo group after one year of treatment. In particular, we found a specific effect of cholinesterase therapy on the BFCS with a treatment difference of −0.44 points compared to a difference of −0.15 detected for total GM. In addition, the treatment effect was observed in those BFCS subregions showing widespread projections to the cortex (NbM), the hippocampus and the entorhinal cortex (Ch1/2). In this regards, our results revealed a relative specificity of tretament for the rostromedial nuclei (Ch1/2) compared to Ch4p/NbM.

It should be noted that the cholinergic basal forebrain, as part of the basomedial limbic belt, is one the most susceptible brain areas for early damage in AD. Cholinergic neurons in the NbM are among the first neurons in which neurofibrillary tangles and Aβ plaques are detected in AD patients^32^. Moreover, a significant *in vivo* atrophy of BFCS in AD patients^20, 33^ as well as in prodromal and preclinical phases of AD^21–23^, particularly in the posterior part of NbM (Ch4p)^24^, has been extensively reported in the recent literature. Notably, *in vivo* BFCS atrophy correlated with cognitive decline in individuals with MCI and AD^22, 34^, and it is assumed to contribute to the mechanisms of brain resilience in cognitively intact elderly individuals^35^. These results support previous evidence describing an involvement of the BFCS in cognitive processes altered in AD, such as memory and attention^36–38^. *In vivo* BFCS atrophy was also found to be associated with cortical brain amyloidosis in AD patients at the pre-symptomatic and pre-dementia stages^27, 28, 39^, thus reflecting a link between cholinergic degeneration and cortical amyloid pathology, as previously reported in neuropathological examination studies^40–42^. A recent analysis emphasized the role of NbM as a crucial pathological target of AD^26^ by describing its early and isolated abnormal degeneration in asymptomatic at-risk individuals for AD. The authors showed how atrophy of the NbM precedes and predicts both memory decline and entorhinal cortex atrophy in relation to AD pathophysiology.

In view of the development of novel therapeutic strategies, the findings presented by Schmitz and colleagues lead to carefully re-examine the prominence of the entorhinal cortex as the structural starting point of the disease and suggest that the BFCS might represent a key therapeutic target for clinical trials aimed at modifying the progression of AD during the early preclinical and potentially reversible or modifiable disease stages. However, to our knowledge, to date no trial has used quantitative BFCS volumetry as primary or secondary endpoint. Few studies examined the association of BFCS volume with treatment response to AChEI in prodromal AD and dementia patients^43, 44^. A first study, conducted in AD dementia patients, showed that the atrophy of the substantia innominate, an anatomic proxy of the BFCS, measured on MRI, predicts response to Donepezil treatment over nine months^43^. However, these effects were not assessed in relation to a Placebo control group. Instead, a study from our group, performed on the hippocampus study population, assessed the significance of hippocampus and BFCS volumes at baseline in predicting cognitive decline and treatment response after one year of Donepezil treatment in comparison to Placebo^44^. Results indicated that both hippocampus and BFCS volumes were poor predictors of treatment response; however, the study sample power was not calculated to detect treatment effects on cognition or functional outcomes^44^. These results highlighted the need to use longitudinal measurements of hippocampus and BFCS to test the treatment effects of Donepezil.

Our results are in line with the ones described in previous clinical trials revealing a biological effect of AChEI treatment in AD dementia patients. In particular, a significant reduction of grey matter and white matter atrophy, as well as metabolic changes were described in AD dementia patients treated with Donepezil compared to Placebo^7–10^. Furthermore, our previous study on prodromal AD patients confirmed the results described in AD dementia patients, showing reduced annual hippocampal rate of atrophy in patients treated with Donepezil compared to the Placebo group^13^. The treated group showed a percentage of annual atrophy reduction equal to 45%. In the current study we found 59% reduction of rate of BFCS atrophy in the treated group. These results suggest a relative higher protective effect of Donepezil on the BFCS compared with the one found in the hippocampus. Additionally, we found an effect of Donepezil in reducing the rate of atrophy in cortical areas involved in the cholinergic circuits^16^. In the same cohort, we found a reduction of whole brain atrophy and ventricular enlargement in prodromal AD participants receiving Donepezil^13^, consistent with earlier findings in MCI individuals^45^. Studies on functional brain imaging reported promising results on the impact of AChEI-based therapy on AD patients showing an increased resting state metabolism in the left prefrontal cortex and a decreased metabolism in the right hippocampus^46^. Furthermore, stabilization of resting- and memory-specific brain activity and connectivity^47, 48^ as well as significant brain activation of the dorsal visual pathway during a location matching task were found in individuals with MCI treated with AChEI^49^.

Our current findings based on quantitative analysis of longitudinal neuroimaging data support the hypothesis that one-year Donepezil treatment at the prodromal AD stage may have a significant positive impact on brain structures involved in the cholinergic circuits, such as the NbM. Furthermore, in the Donepezil group, our results highlighted the presence of reduced atrophy rate in the Ch1/2 subregions, representing the primary source of cholinergic projections to the hippocampus and entorhinal cortex^26, 50^. Two previous trials showed reduced hippocampal atrophy after Donepezil treatment in comparable target populations^11, 13^. Consequently, Donepezil may induce a specific beneficial pathophysiological effect on brain structures involved in the hippocampal cholinergic circuit. This reflection is in line with previously published evidence suggesting that AChEI therapy probably delays the progression of the disease by reducing the accumulation of Aβ plaques in the brain^5^. In addition to cholinergic system enhancement, there is evidence that Donepezil may promote Nerve Growth Factor (NGF) activity and potentiate NGF-induced neurite outgrowth via extracellular signal-regulated kinase (ERK) activation in PC12 cells^51^. These results suggest that Donepezil may promote neuronal differentiation and this effect could slow the degeneration processes fostering the recovery of higher neuronal functions. Furthermore, several studies suggest a relationship between cholinergic neurotransmission and β-amyloid peptide (Aβ) that may act as a physiologically active neuromodulator^52^. The scenario of chronic inflammation in the brain, generated by the imbalance between Aβ production and clearance, induces the production of a series of proinflammatory cytokines, chemokines, macrophage inflammatory proteins, leukotrienes, reactive oxygen species, and nitric oxide^53–55^. The neuroinflammatory cytokines may not only contribute to neuronal death, but they might also affect the recognized neurodegenerative pathways such as amyloid precursor protein (APP) processing and tau hyperphosphorylation^56^. Studies using Donepezil have also shown that Donepezil does not act exclusively at the level of acetylcholine, but has potent anti‐inflammatory properties in AD patients, in a tauopathy mouse model and in lipopolysaccharide (LPS)‐treated animals^57^. These findings suggest a potential inhibitory role of Donepezil in neuronal death and cognitive decline by repressing oligomeric Aβ‐triggered inflammatory pathways in microglia^57^.

Further analyses on phenotypically and clinically well-defined populations, supported by *in vivo* evidence of AD pathophysiology, are needed in order to substantiate our findings.

A former investigation examining longitudinal BFCS atrophy in very mild AD patients based on the same methodology showed a BFCS APC of 2.9%^23^. The higher APC value than the one found in our Placebo group may be due to differences in the clinical population specifically selected. Indeed, participants selected for the present trial mostly represent prodromal AD patients at a particularly early stage, as defined by the amnestic syndrome of the hippocampal type.

Our study has a number of limitations that might reduce its generalizability. First of all, the initial study design was not intended for use in a *post-hoc* analysis. The study protocol did not collect any information with reference to core feasible pathophysiological biomarkers of AD, race and ethnicity characteristics, or lifestyle. Lifestyle habits, such as nutrition, hydration, physical activity, and bilingualism, showed a significant effect on brain morphology in elderly individuals^58–60^. Moreover, no sufficient data on *APOEε4* genotype were collected to exclude the hypothesis that our results were not significantly affected by the *APOE ε4* genetic variant that seems to have an impact on the biological effect of Donepezil in the amyloid precursor protein metabolism in AD dementia patients^61^. Finally, the examined follow-up period was not long enough to determine the incidence of incipient AD dementia in each group.

In conclusion, to our knowledge this is the first randomized clinical therapy trial using the BFCS as outcome. Overall, our findings substantiate the hypothesis that Donepezil treatment during the prodromal AD stage may preserve decline of brain morphology of structures belonging to the cholinergic circuit, thus re-opening the question whether Donepezil has substantial biological properties and should be still categorized as a mere “symptomatic” drug. Moreover, our findings support the assumption that BFCS atrophy may represent a robust, reliable and feasible candidate surrogate outcome in pre-dementia AD clinical trials. Our results furthermore, are in line with recent findings from Schmitz and colleagues describing a central and early involvement of basal forebrain atrophy during the pathophysiological process of AD^26^. In the light of these findings, the effect of cholinergic therapy should then be applied and assessed in a preclinical population of amyloid positive individuals, in order to test the power of cholinergic drugs in preventing AD.

Cholinergic structural changes begin during subclinical stages of AD and are accompanied by several other components of AD pathophysiology leading to AD dementia. For these reasons, further studies combining the *in vivo* measurement of different pathophysiological mechanisms of AD, such as neurofibrillary tangles and amyloid plaque deposition measured by positron emission tomography, as well as white matter cholinergic networks measured by advanced tractographic methods, are needed to support data on the relative specificity of Donepezil effect on the cholinergic system in the brain.

## Methods

### Study Design

Multi-centre double-blind, randomized, placebo-controlled trial with a treatment period of 12 months. The study started in November 2006 and was concluded in August 2010 (NCT00403520), for details on the selection of population, randomisation and sample size calculation please see our previous publication by Dubois and colleagues^13^. Patients were randomly assigned to either active or Placebo treatment group (1 capsule of 5-mg Donepezil daily for Weeks 0 to 6, then 2 capsules of 5-mg Donepezil [i.e., 10 mg] daily from Week 6 to Month 12 for double-blind treatment; or 1 placebo capsule daily for Weeks 0 to 6, then 2 capsules daily from Week 6 to Month 12 for double-blind treatment, respectively). The study protocol was approved by the institutional review board from each site and by the Ethic Committee of the Coordination Centre at La Pitié-Salpêtrière Hospital, Paris, France (Reference Number: E2020-E033-41, Ethic Committee Approval: November 8, 2006). The study was performed strictly in accordance with the approved guidelines and regulations. An informed consent was obtained from all subjects. The original primary efficacy outcome of the trial was the annualized percentage change (APC) of total hippocampal volume measured by an automated segmentation method^62^. Since the BFCS is one of the main brain regions rich of cholinergic neurons and early involved in the AD pathophysiology, in this *post-hoc* analysis, we explored the effect of Donepezil on BFCS APC.

### Patients

Details on patient characteristics have been previously described^13^. Briefly, a total of 332 patients were screened within the national network of Memory Resources and Research Centres (CMRR) consisting of 28 French regional university expert centres with neurologists, geriatricians, neuropsychologists, biological and neuroimaging resources in each centre. Inclusion criteria were: 1) more than 50 years of age; 2) a progressive hippocampal amnestic syndrome, denoting prodromal AD, was defined by Free Recall score ≤17 or Total Recall score <40 on the Free and Cued Selective Reminding Test (FCSRT); and 3) no dementia with a CDR stage of 0.5 and preserved global cognition and functional performance. Subjects who met the eligibility criteria were enrolled in the randomization phase beginning with Visit 1. In the present study we considered exclusively the per-protocol population (173 subjects) who performed MRI at baseline and at the end of the treatment.

### Neuropsychological assessment

At baseline and follow-up visits, patients underwent a baseline MRI scan and a cognitive evaluation including: the Alzheimer’s Disease Assessment Scale-cognitive subscale, MCI version (ADAS-COG-MCI)^20^, Mini-Mental State Examination (MMSE), Modified Isaacs test, California Verbal Learning Test (CVLT)^21^, Trail Making Tests (TMT) A and B^22^, and the Benton Test^23^.

### MRI Acquisition

Brain MRI scans were acquired in each centre at baseline and at the end of treatment period. All the MRI were performed using 1.5 Tesla or 3 Tesla MRI scanners qualified by the central MRI analysis core at the Cogimage team, Centre de Recherche de l’institut du Cerveau et de la Moelle Épinière (CRICM). A standardized protocol of acquisition for the 3D T1-weighted images was applied in each site. The 3D T1-weighted scan parameters for scans at 1.5 T were: TR = 10 ms, TE = minimum, flip angle: 10, PREP time: 600, bandpass: 12.50 khz, axial orientation; 1.3mm slice thickness, contiguous (124 slices), Imaging matrix size: 256 × 256, Field of view: 240 × 240; and for scans at 3 T: TR = 3.9 ms, TE = 2100 ms, flip angle: 15, PREP time: 1100 ms, sagittal orientation with 1 mm slice thickness, contiguous (144 slices), Acquisition matrix size: 256 × 192, Field of view: 256 × 192. The expert neuroradiologist in charge of quality control verified artifacts of movements, ringing, wrap around and metal artifacts. Moreover they attested that the sequences were acquired according to protocol parameters. Due to gross MRI artefacts or MRI quality issues, which might interfere with data analysis measurement of BFCS volume, 10 MRI images were excluded. The final sample considered for the study was composed of 163 subjects (Placebo N = 88 and Donepezil N = 75).

### MRI processing

Main processing steps and computational analyses employed in the present study followed the longitudinal processing stream implemented in SPM12^63^, adapted to the analysis of serial MRI scans used for BFCS volume^23^. MRI data were processed by the statistical parametric mapping (SPM8 and SPM12, Wellcome Trust Center for Neuroimaging) and the VBM8-toolbox (http://dbm.neuro.uni-jena.de/vbm/) implemented in MatLab R2013a (MathWorks, Natick, MA). First, we applied the symmetric diffeomorphic registration for longitudinal MRI developed in SPM12^63^. In particular, this algorithm rests on an intra-subject modelling framework that combines nonlinear diffeomorphic and rigid-body registration and further corrects for intensity inhomogeneity artefacts. The optimization is realized in a single generative model and provides internally consistent estimates of within-subject brain deformations that may have occurred during the study period. The registration model creates an average T1-image for each subject and the corresponding deformation fields for every individual scan. Then, images were segmented into grey matter (GM), white matter (WM) and cerebrospinal fluid (CSF) partitions using the tissue prior free segmentation routine of the VBM8-toolbox. All GM maps were visually inspected for segmentation. Afterward, nonlinear inter-subject image registration was performed on the individual average GM and WM tissue maps using DARTEL in SPM12. This involves iteratively matching all the selected images to a template generated from their own mean. Then, all native space average GM maps were scaled by the respective Jacobian determinants (baseline and follow-up) obtained from the longitudinal registration, and the resulting images were finally warped to the group-specific spatial normalization template. Finally, individual GM volumes of the BFCS at baseline and follow-up were extracted automatically by summing up the modulated GM voxel values within a stereotactic mask of the BFCS. Generation of the BFCS mask was based on combined post-mortem MRI and histology of an autopsy brain and has been described in detail elsewhere^24^. Briefly, the BFCS and its subregions were identified from digital images of histological sections of a post-mortem brain and transferred into corresponding slices of an *in-situ* acquired MRI scan, which was used for high-dimensional spatial normalization into MNI standard space. Histologic delineation of the BFCS and its subregions followed Mesulam’s nomenclature^64, 65^, according to which the BFCS is separated into 4 principal groups of cholinergic cells: Ch1 and Ch2 denote the cholinergic cells associated with the medial septal nucleus, and the vertical limb of the diagonal band of Broca (Ch1/Ch2); Ch3 belongs to the horizontal limb of the diagonal band of Broca, and Ch4 describes the cholinergic cells of the NbM^65^. The NbM is the largest cholinergic nucleus of the BFCS and can be considered as the sum of its subdivisions: anterior to intermediate (Ch4a-i), anterior lateral/nucleus subputaminalis (Ch4al/NPS) and posterior (Ch4p) subregions.

### Statistical Analysis

Demographic, clinical and neuropsychological features were compared between groups (Placebo vs Donepezil). Chi-square (two-tailed) test was performed on categorical variables, while the one-way analysis of variance (ANOVA) two-tailed test was utilized for continuous variables. Baseline GM and BFCS measures normalized to total intracranial volume were compared between groups using One-way ANOVA two-tailed test.

Annualized Percentage Change (APC) of GM, BFCS and its subregions was computed as follows:

$${APC}=\frac{change\,from\,baseline}{value\,at\,baseline}\times \frac{365}{MRI\,delay}\times 100$$

A General Linear Model including age, gender, MRI field strength (1.5 T or 3 T) and MRI scanner manufacturer (Philips, GE Healthcare or Siemens) was performed on the APC of the GM, BFCS and its subregions. In particular, in the whole NbM and its posterior nucleus (Ch4p) considered as the most susceptible to AD pathology^21, 23, 25, 66^, and in the medial septum and the vertical limb of the diagonal band of Broca (Ch1/2), which provide the main cholinergic innervation of the hippocampus. The BFCS, its subregions and the GM volumes were supplemented analysed by a linear mixed-effects model with patients as random effects and controlling for: age, gender, MRI field strength and MRI scanner manufacturer, to compare the differences in the volumes changes between Placebo and Donepezil patients over time. Statistical analyses were performed using SPSS v.22.00 and R 3.3.2.

## Electronic supplementary material

Supplementary Figure 1
