# Supplementary material for: Reduced basal forebrain atrophy progression in a randomized Donepezil trial in prodromal Alzheimer’s disease
Source: Sci Rep. 2017 Sep 15;7:11706. doi: 10.1038/s41598-017-09780-3 (PMC5601919; doi:10.1038/s41598-017-09780-3)

Reduced basal forebrain atrophy progression in a randomized Donepezil trial in prodromal Alzheimer's disease

Enrica Cavedo, Michel J. Grothe, Olivier Colliot, Simone Lista, Marie Chupin, Didier Dormont , Marion Houot, Stephane Lehéricy, Stefan Teipel, Bruno Dubois, Harald Hampel
and the “Hippocampus Study Group”**

Supplementary Figure 1. Scatter plots describing the distribution of Grey Matter and Basal Forebrain Cholinergic System volumes in each treatment group stratified by MRI field strength at baseline and follow-up scans.

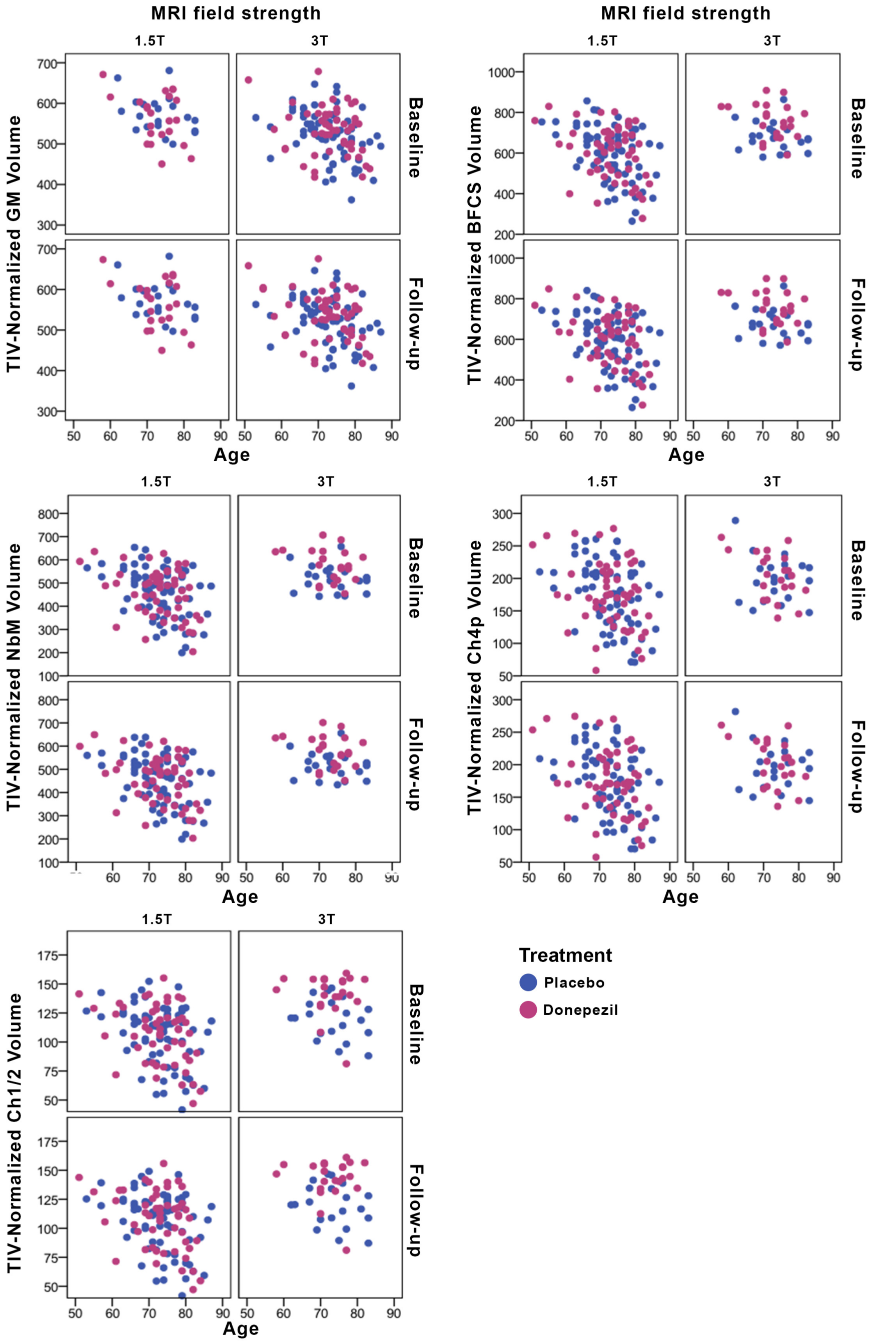

Supplement: Supplementary file 1 — Supplementary Figure 1 [file 41598_2017_9780_MOESM1_ESM.pdf]
